# Cadherin-12 enhances proliferation in colorectal cancer cells and increases progression by promoting EMT

**DOI:** 10.1007/s13277-015-4555-z

**Published:** 2016-01-14

**Authors:** Junjun Ma, Jingkun Zhao, Jun Lu, Puxiongzhi Wang, Hao Feng, Yaping Zong, Baochi Ou, Minhua Zheng, Aiguo Lu

**Affiliations:** 1 Shanghai Minimally Invasive Surgery Center, Ruijin Hospital, Shanghai Jiaotong University School of Medicine, Shanghai, People’s Republic of China; 2 Shanghai Institute of Digestive Surgery, Shanghai, People’s Republic of China; 3 Department of Surgery, Munich University, Munich, Germany; 4 Department of General Surgery, Hangzhou First People’s Hospital, Hangzhou, Zhejiang People’s Republic of China

**Keywords:** Cadherin-12, Colorectal cancer, EMT, Metastasis, Snail, MCP1

## Abstract

Cadherin-12 (CDH12) is a subtype of N-cadherin family. In this study, we investigated the expression of CDH12 and the role of CDH12 in prognosis of colorectal cancer (CRC) patients. In addition, we observed the influence of CDH12 on proliferation and progression of CRC cell lines. By using immunohistochemical staining, we analyzed CRC samples and adjacent non-tumor tissues collected from 78 patients who underwent laparoscopic surgery in Shanghai Minimally Invasive Center, China. Statistical analyses were used to analyze relationship between CDH12 and tumor features. Kaplan-Meier method was used to analyze patients’ survival. Proliferation ability of CRC cells was tested by CCK-8 assay, and transwell assays were performed to detect migration and invasion ability. Western blot assay was performed to investigate epithelial-mesenchymal transition (EMT) variants. We found that expression of CDH12 in tumor tissue was higher than in adjacent normal tissue. High expression of CDH12 was associated with tumor invasion depth and predicts poor prognosis of CRC patients. Ectopic/repressing expression of CDH12 increased/decreased the proliferation and migration ability of CRC cells. CDH12 is able to increase cancer cell migration and invasion via promoting EMT by targeting transcriptional factor Snail. These findings may conclude that CDH12 may act as a predictor in CRC patients’ prognosis and an oncogene in CRC cell proliferation and migration. CDH12 may influence CRC cell progression through promoting EMT by targeting Snail. In addition, CDH12 is promoted by MCP1 through induction of MCPIP.

**Electronic supplementary material:**

The online version of this article (doi:10.1007/s13277-015-4555-z) contains supplementary material, which is available to authorized users.

## Introduction

Colorectal cancer (CRC) is one of the most prevalent cancer types worldwide and leads to the third death of cancer-related patients. The major cause of deaths for CRC patients is distant organ metastasis especially liver metastasis [1]. Colorectal cancer has the propensity to colonize the liver and leads to a poor prognosis in the patient. Although metastasis-related genes have been mostly clarified in CRC [2], metastasis is a complex process and has not been fully understood.

The epithelial-mesenchymal transition (EMT) is a dynamic cellular process that is essential for the development of metastatic disease [3]. During EMT, tumor cells with epithelial characteristics transition to those with mesenchymal characteristics through modulation of cell polarity and adhesion [4] which contributes to cancer progression [5]. The destruction of cadherin junction especially the downregulation of epithelial cadherin (E-cadherin) has been regarded as an important marker of EMT. The importance of cadherin in EMT has been verified in many types of human tumor [6, 7]. In epithelial tumor, immunostaining to the E-cadherin protein demonstrates intimate relation between decrease of this protein and tumor invasiveness [6]. Prominent regulation of E-cadherin is related to series of transcription factors such as Snail family which has a common structure of zinc finger and consists of Snail 1 (Snail), Snail 2 (Slug), Snail 3 (Smuc), and the basic helix-loop-helix (bHLH) transcription factor twist and E12/E47. Regulation factors above can bind to the E-boxes of E-cadherin promoter and thus suppress the transcription of E-cadherin in response to upstream signaling [8]. Recently, it has been accepted that EMT is an important and complex phenomenon which determines the aggressiveness of colon cancer [9]. Thus, to elucidate the molecular mechanisms of EMT may favor the progression of colon cancer research.

Cadherin-12 (CDH12) is a subtype of neural cadherin (N-cadherin) that belongs to cadherin family which is a flock of membrane-spanning Ca^2+^-dependent homophilic adhesion receptors [4]. CDH12 is located in chromosome 5 p14.3 and codes peptide with 88-kD molecular mass including 794 amino acid residues. CDH12 is ubiquitously expressed in a wide range of cell types. Relative studies verified that CDH12 plays a significant role in the progression of non-small-cell lung cancer and salivary adenoid cystic carcinoma [10, 11]. However, role of CDH12 in colon cancer is still largely unknown. In this article, we presented the clinical significance of CDH12 in CRC patients’ specimens and elucidated its preminent influence on colon cancer cell proliferation as well as invasion. In addition, we found that CDH12 promotes CRC cell metastasis by promoting EMT via targeting transcriptional factor Snail. Moreover, monocyte chemotactic protein 1 (MCP1) is able to modulate CDH12 expression through induction of MCP1-induced protein (MCPIP). These findings may partially reveal possible molecular mechanisms of CDH12 promoting migration and invasion in CRC cells.

## Patients, materials, and methods

### Clinical specimen microarray and immunohistological analysis

Seventy-eight pairs of CRC tissue and adjacent normal tissue were included in our study. All the patients were diagnosed specifically as CRC and treated with laparoscopic surgery in Minimally Invasive Surgery Center, Ruijin Hospital, Shanghai Jiaotong University, from 2010 to 2011. The collection of specimens was authorized by Ethical Committee of Ruijin Hospital. All the patients who had received preoperative treatment such as radiation or chemotherapy, those who had lesions such as regional or organ unresectable metastasis, were excluded. The patients include 43 males and 35 females. Pathological staging of CRC tumor was performed in accordance to the TNM classification provided by World Health Organization [12]. Fresh specimens were immediately fixed by 10 % formaldehyde after dissection and embedded with paraffin. Tissue microarrays were manufactured by Outdo Biotechnology Corporation. Immunohistological staining of tissue microarray was performed as the following: after being dewaxed at 60 °C and hydrated gradiently, the microarray was antigen retrieved by microwaving in citrate buffer (10 mM citric acid, pH 6.0), blocked in 5 % animal serum, and stained for 2 h at 37 °C by primary antibody. Secondary biotinylated anti-rabbit antibody was used to stain the microarray for 10 min at 37 °C. Microarrays were visualized by DAB, and the nucleus was counterstained by hematoxylin. Microscope was used to acquire pictures which were analyzed by two pathologists independently. Staining results were evaluated following the scoring criteria described by Shimazui T et al. [13, 14] as we have provided previously. Expression of CDH12 was scored as positive (+), heterogeneous (+/−), or uniformly negative (−) when the CDH12 was uniformly expressed, heterogeneously expressed, or not expressed in the tissue, respectively.

### Cell culture

Human CRC cell lines used in our study were purchased from American Type Culture Collection. The cell lines were preserved by Shanghai Digestive Surgery Institute. SW620 and SW1116 cell lines were cultured by Leibovitz’s L-15 medium, and HCT116 was cultured by McCoy’s 5A medium in 37 °C, 5 % CO_2_ incubator for use.

### Protein extraction and Western blot analysis

Total protein was extracted using RIPA (Solarbio) at a 70–80 % confluence of cells. Protein lysates were reacted for 30 min on the ice and centrifuged for 15 min at 13,000 rpm. The supernatant was collected for Western blot analysis. The concentration of protein lysates was tested via bicinchoninic acid (BCA) reagent using BCA Protein Assay Kit (Thermo Scientific) by measuring optical density (OD) 562. Western blot analysis was performed as previous description. In this experiment, primary antibodies were rabbit polyclonal anti-CDH12 antibody (Abcam), 1:100; rabbit polyclonal anti-EMT marker antibodies (including E-cadherin, N-cadherin, beta-catenin (β-catenin), Snail, and Slug), 1:1000 (Cell Signaling Technology); and mouse polyclonal anti-GAPDH antibody, 1:5000. Fluorescence-labeled secondary antibody was used to visualize results by incubating for 2 h at room temperature. LI-COR Odyssey infrared fluorescence scanner was used to capture the images.

### Short hairpin RNA transfection

Green fluorescent protein (GFP)-labeled short hairpin RNA (shRNA) targeting CDH12 was purchased from Gene-Pharma Corporation. Of the cells, 3 × 10^5^ was seeded into each chamber 6-well plate 1 day before transfection experiment. Lipofectamine^™^ 2000 (Invitrogen Corporation) was used to mediate shRNA against CDH12 into cells after 70 % confluence. According to transfection instruction, shRNA targeting non-homologous gene to CDH12 was performed as control. Forty-eight hours after transfection, cells were harvested and the protein levels of the targeted genes were assessed by Western blot. Antibiotics G418 was used to screen stable CDH12 expression cell clones. Interfering effect was evaluated by Western blot. Targeting site is listed below.

- shCDH12-1:5′-CACCGCTGGGCAACAATTCTCCTTTTCAAGAGAAGGAGAATTGTTGCCCAGCTTTTTG-3′ (sense sequence);
- shCDH12-2:5′-CACCGCGCAGTATAATTTCTCCATACTCGAGTATGGAGAAATTATACTGCGCTTTTTG-3′ (sense sequence);
- shCDH12-3:5′-CACCCGGTCACATTTCCAACGTGTTCTCGAGAACACGTTGGAAATGTGACCGTTTTTG-3′ (sense sequence);
- Negative control 5′-CACCGTTCTCCGAACGTGTCACGTCAAGAGATTACGTGACACGT TCGGAGAATTTTTTG-3′ (sense sequence).

### Lentivirus stable transfection

The construction of lentivirals with plasmids containing GFP and CDH12 total gene sequences or negative control (NC) gene sequences marking with anti-antibiotic gene were purchased from Novobio Corporation. Lentivirus transfection was performed according to the manufacturer’s instruction. CDH12 protein expressions were examined by Western blot.

### Cell viability assay

About 2000 cells were seeded in each well of 96-well plate and cultured in 37 °C, 5 % CO_2_ incubator. Three groups (shCDH12 group, shNC group, and Mock group for SW620/shCDH12 and CDH12 group, Vector group, and Blank group for HCT116/CDH12) were designed with six copies each group. The viability of cells was detected at 0, 24, 48, 72, 96, and 120 h. At the test point, 10-μL Cell Counting Kit-8 (CCK-8) was added into each well and the plate was incubated at 37 °C for 2 h followed by OD detection using spectrophotometer.

### Plate clone formation assay

Plate clone formation assay was performed as the following: cells were resuspended to get the single-cell suspension, and then, cells were seeded in 6-well plates at a density of 1500 cells per well. After macroclones (larger than 5 mm) were formed, cell clones were fixed with methanol and stained with 1 % crystal violet.

### Cell invasion and migration assays

Cell invasion and migration assays were performed by using transwell chamber (8 mm, 24-well format; Corning) which was coated with or without diluted Matrigel (BD Biosciences). Assays were performed as the following: 200-μL serum-free medium containing 3 × 10^5^ cells was placed in the upper chamber and 600-μL culture medium with 10 % serum was added into the lower chamber as attractant. The chamber was cultured in 37 °C, 5 % CO_2_ incubator for 24 h. Cotton swab was used to remove reduced cells in the top chamber, and then, the chambers were fixed with methanol. Cells outside the inserts were stained with 1 % crystal violet for 30 min. Cells in five random fields of cells were photographed under microscope, and the results are calculated as means ± SD.

### Immunofluorescence assay

For immunofluorescence assays, 40,000 cells were cultured in each cell of EZ slides (Millicell EZ SLIDE 8-well glass, Millipore). After being incubated for 24 h, each well was fixed in 4 % paraformaldehyde for 15 min. Cells were permeabilized with 0.1 % Triton X-100 for 15 min at room temperature, washed with phosphate-buffered saline (PBS) for three times, and blocked with PBS containing 5 % (*w*/*v*) bovine serum albumin (BSA) 1 h at room temperature. Cells were treated with anti-E-cadherin antibody (1:50, Abcam) and anti-β-catenin antibody (1:50, Abcam) and incubated overnight at 4 °C. A negative control (without primary antibody) was included on every slide. In the second day, each well were washed with PBS and incubated with iFluor^™^ 594 goat anti-mouse antibody (AAT Bioquest, USA) for 2 h at 37 °C. After being washed with PBS, diamidino-2-phenylindole (DAPI; Santa Cruz) was used to counterstain nucleus. Results were obtained from fluorescence microscopy (Olympus, Japan).

### Nude mouse experiment

Animal experiment obeyed the ethical code and recommendation issued by Chinese Animal Community. Four-week-old male BALB/C nude mice were purchased from the Institute of Zoology, Chinese Academy of Sciences of Shanghai. In this experiment, 10 nude mice were included in each group. Of shCDH12, shNC, and Mock, 2 × 10^7^ SW620 cells/ml were injected into nude oxter, and 2 × 10^7^ HCT116 cells/ml of CDH12, Vector, and Blank were injected into nude oxter. After 30-day feeding, nude mice were killed by cervical decapitation and cancer nodules were excised to perform further morphological analysis. The size of tumor was calculated as the following: a*b^2/2; among this formula, “*a*” represents the long diameter of tumor and “*b*” represents the short diameter of tumor.

### Statistical analysis

All the statistical analyses were performed by SAS 8.0 statistical software. The difference of CDH12 expression between tumor tissue and adjacent normal tissue was examined by Cochran-Maantel-Terpstra test. Chi-squared test and Fisher exact probability method were used to analyze the relationship between CDH12 expression level and clinical features. Survival probabilities were calculated using Kaplan-Meier method; differences between two groups were determined using the log-rank test. Multiple comparisons were performed by one-way analysis of variance. *P* < 0.05 was considered to be statistically significant.

## Results

### Expression of CDH12 in CRC patients is correlated with tumor invasion depth and predicts poor prognosis of CRC patients

To evaluate CDH12 expression in CRC tissue, we used immunohistochemistry (IHC) staining to test tissue microarray obtained from 78 CRC patients. The pathological and statistical results were shown in Supplementary Fig. 1 and Table 1. We evaluated the IHC results and cases which demonstrate that uniformly positive staining of CDH12 was regarded as “CDH12 high expression” in the statistical analysis. Reversely, those which demonstrate heterogeneous or uniformly negative staining of CDH12 were evaluated as “CDH12 low expression.” According to these criteria, of the 78 CRC specimens, 53 tumor tissue samples exhibited a high expression of CDH12 versus 25 cases in adjacent normal tissue (*P* < 0.01). In addition, 15 cases expressed heterogeneously and 10 cases expressed uniformly negative which were evaluated as CDH12 low expression (Table 1). CDH12 was mainly expressed along cell membrane (Supplementary Fig. 1e, f). Then, we made a statistical analysis between staining results and clinical materials of patients which were presented in Table 1. Statistical results were shown in Tables 2 and 3; low expression of CDH12 was predominantly associated with tumor invasion depth (*P* = 0.02) and lymph node metastasis (*P* = 0.04) but has no relationship with patients’ age (*P* = 0.88), tumor size (*P* = 0.90), tumor histology (*P* = 0.60), and distant colonization (*P* = 0.36) (Table 3). Therefore, CDH12 may be involved in CRC invasion into mesenchymal tissue and lymph node metastasis.

**Table 1:** Comparison of CDH12 expression in tumor and normal tissue

Parameters	Number	CDH12 (−)	CDH12 (+/−)	CDH12 (+)	*P* value
Tumor tissue	78	16	9	53	<0.01
Normal tissue	78	47	6	25

**Table 2:** Clinicopathological data

Parameters	Case number
Gender	
Male	43 (55.1 %)
Female	35 (44.9 %)
Age	
≤65	47 (60.3 %)
>65	31 (39.7 %)
Tumor location	
Right hemicolon	24 (30.8 %)
Transverse colon	3 (3.8 %)
Left hemicolon	7 (9.0 %)
Sigmoid colon	22 (28.2 %)
Rectum	22 (28.2 %)
Tumor size	
≤5 cm	43 (55.1 %)
>5 cm	35 (44.9 %)
Tumor histology	
Tubular adenocarcinoma	65 (83.3 %)
Mucinous adenocarcinoma	12 (15.4 %)
Papillary adenocarcinoma	1 (1.3 %)
Pathological stage	
I	12 (15.4 %)
II	29 (37.2 %)
III	32 (41.0 %)
IV	5 (6.4 %)

**Table 3:** Comparison between CDH12 expression and clinicopathologic variables in CRC patients

Classification	Group	CDH12 (−)	CDH12 (+/−)	CDH12 (+)	*P* value
Gender	Male	9	5	29	0.99
	Female	7	4	24	
Age	≤65	10	6	31	0.88
	>65	6	3	22	
Tumor location	Right hemicolon	5	3	16	0.30
	Transverse colon	0	1	2	
	Left hemicolon	4	1	2	
	Sigmoid colon	3	3	16	
	Rectum	4	1	17	
Tumor size	≤5 cm	8	5	30	0.90
	>5 cm	8	4	23	
Tumor histology	Tubular	15	8	42	0.60
	Mucinous	1	1	10	
	Papillary	0	0	1	
TNM stage	T_1_	3	1	6	0.02
	T_2_	7	2	7	
	T_3_	5	2	22	
	T_4_	1	4	18	
	N_0_	11	4	20	0.04
	N_1_	4	2	19	
	N_2_	1	3	14	
	M_0_	16	8	49	0.36
	M_1_	0	1	4	
Pathological stage	I	1	2	9	0.33
	II	10	4	15	
	III	5	2	25	
	IV	0	1	4	
CEA	≥5.0	2	3	10	0.76
	<5.0	14	6	43	

**Fig. 1:**
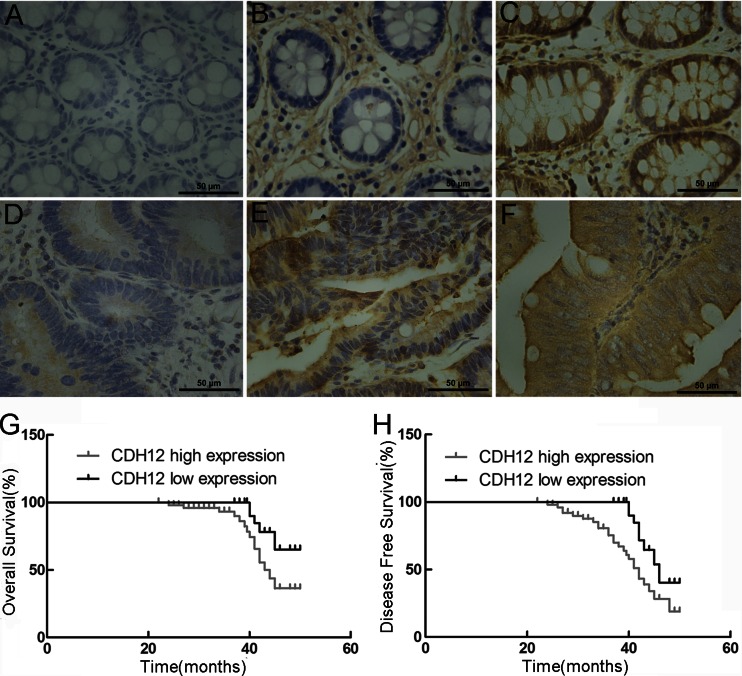
Immunohistochemical staining results of CDH12 in tumor tissues and adjacent normal tissue; Kaplan-Meier survival curves for OS and DFS. **a** Uniformly negative staining in adjacent normal mucosa, **b** heterogeneous staining in adjacent normal mucosa, **c** uniformly positive staining in adjacent normal mucosa, **d** uniformly negative staining in CRC tumor tissues, **e** heterogeneous staining in CRC tumor tissues, and **f** uniformly positive staining in CRC tumor tissues. Magnification 400×. **g** CDH12 high expression indicates undesirable OS and **h** CDH12 high expression indicates unfavorable DFS. *P* < 0.05, both in OS and DFS

In addition, to further evaluate the clinical prognostic significance of CDH12, Kaplan-Meier 5-year survival curve was used to evaluate the relation of CDH12 expression in CRC tissues with the survival time of CRC patients. The Kaplan-Meier survival analysis showed that patients with high CDH12 expression (IHC staining shows uniformly positive) had longer 5-year overall survival (OS) and disease free survival (DFS) than patients with low expression (IHC staining shows uniformly negative and heterogeneous intensity) (*P* < 0.05; Supplementary Fig. 1g, h and Fig. 1).

### Expression of CDH12 in CRC cell lines

**Fig. 2:**
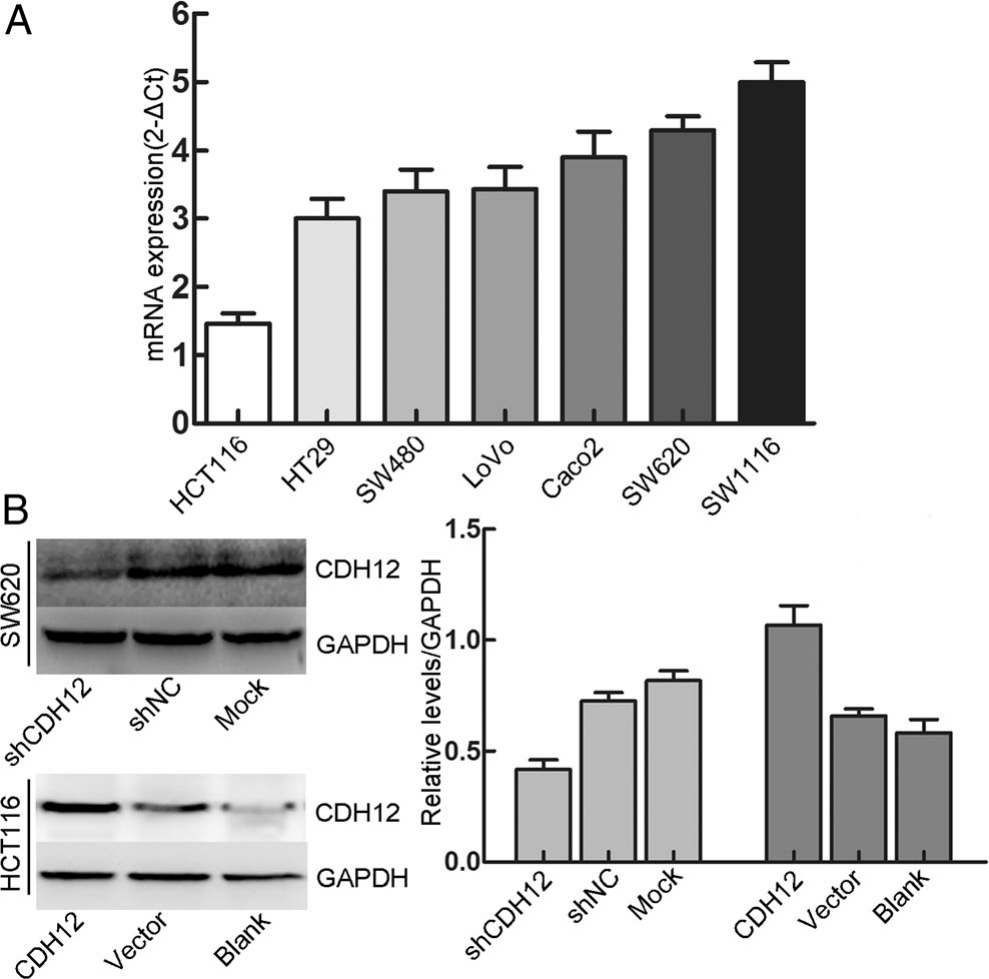
Expression of CDH12 in CRC cell lines. **a** CDH12 expression in CRC cell lines detected by qRT-PCR. **b** *Upper panel* effect of shCDH12 on CDH12 expression in SW620 cells compared with shNC which was transfected into scrambled shRNA and Mock which was transfected into Lipofectamine 2000. *Lower panel* ectopic expression of CDH12 in HCT116 transfected with lentivirus-CDH12. Vector and Blank groups were transfected into lentivirus NC and nothing, respectively. *Column graph* shows the relative *grey level* of each band compared with GAPDH

We analyzed the expression of CDH12 in seven colon cancer cell lines by using qRT-PCR. CDH12 was expressed in all these cell lines and especially high in SW1116 and SW620 but low in HCT116 (Fig. 2a). Considering the ability of tumor formation in nude mice, we have chosen SW620 and HCT116 as our experimental objects. After screening of shRNA, we used the most efficient interfering shRNA to transfect SW620 and downregulated the expression of CDH12. Interfering effect was identified by Western blot (Fig. 2b). In addition, CDH12 lentivirus was used to transfect into HCT116 to upregulate the expression of CDH12. The results showed that the expression of CDH12 was significantly increased in HCT116 compared with control groups (Fig. 2b).

### High levels of CDH12 can promote cell proliferation in CRC

**Fig. 3:**
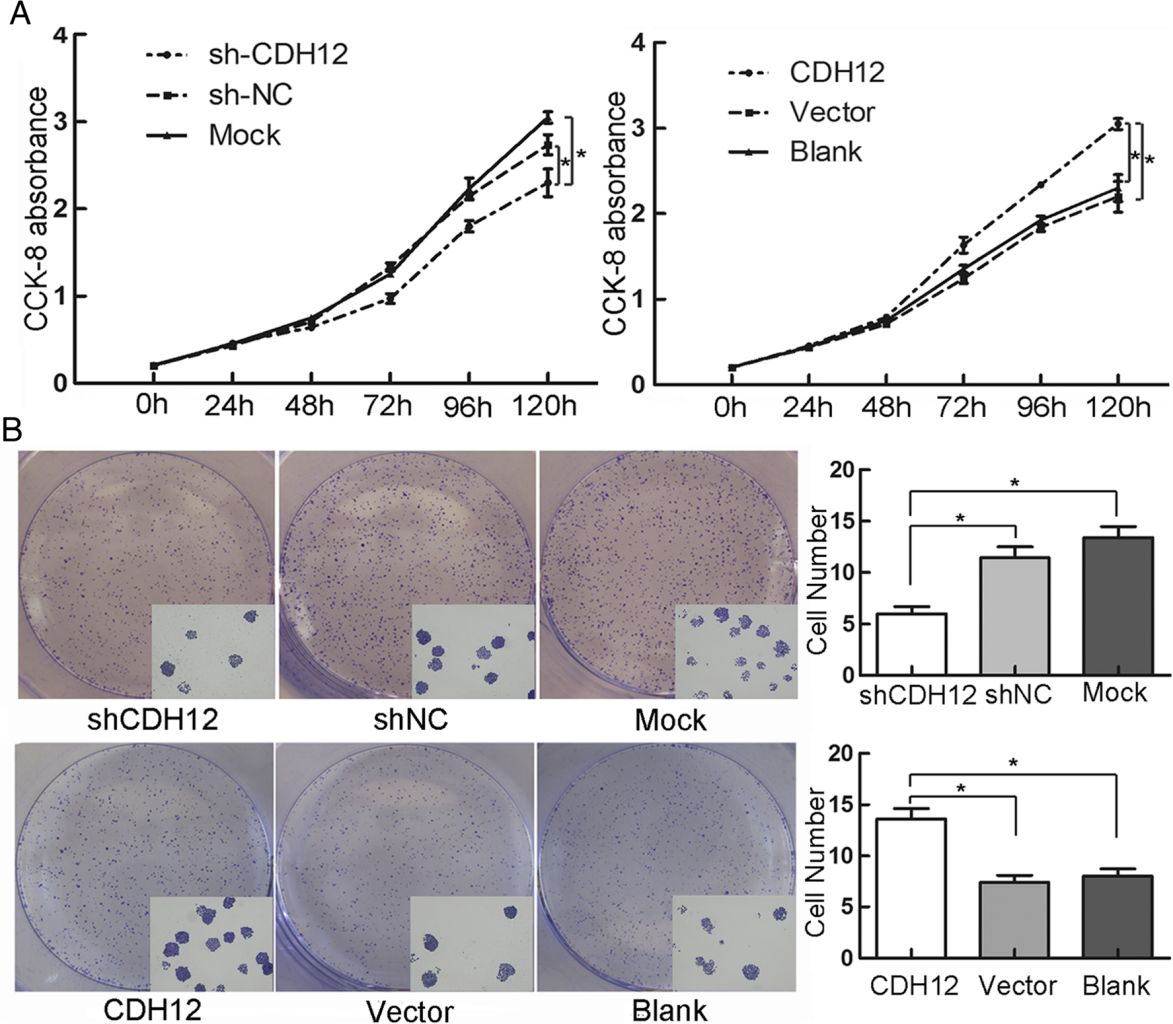
**a** Proliferation curve for SW620 cells with low CDH12 expression whose proliferation ability was inhibited compared with controls (*left panel*) and HCT116 cells with high-expression CDH12 whose proliferation ability was promoted (*right panel*). **b** Clone formation quantities were less or more in CDH12 low-expression group (SW620, *upper panel*) or in high-expression group (HCT116, *lower panel*) compared with controls. *P < 0.05

To verify if CDH12 is able to influence the proliferative function of CRC cell lines, we performed CCK-8 proliferation assay. Thus, we constructed CDH12 stable knockdown clone, SW620/shCDH12 cells, and the negative control clone, SW620/shNC cells. In addition, upregulated clone HCT116/CDH12 and HCT116/Vector were also constructed, respectively, with lentivirus. CCK-8 was used to test cell proliferation ability after enforcing or downregulating CDH12 expression. Proliferation abilities of cancer cells were examined at six time points (0, 24, 48, 72, 96, and 120 h). Compared with the control groups, cells expressing high levels of CDH12 (HCT116/CDH12) showed significantly high proliferational potential (*P* < 0.05; Fig. 3a); however, cells with lower CDH12 level (SW620/shCDH12) presented significantly decreased proliferation ability (*P* < 0.05; Fig. 3a).

**Fig. 4:**
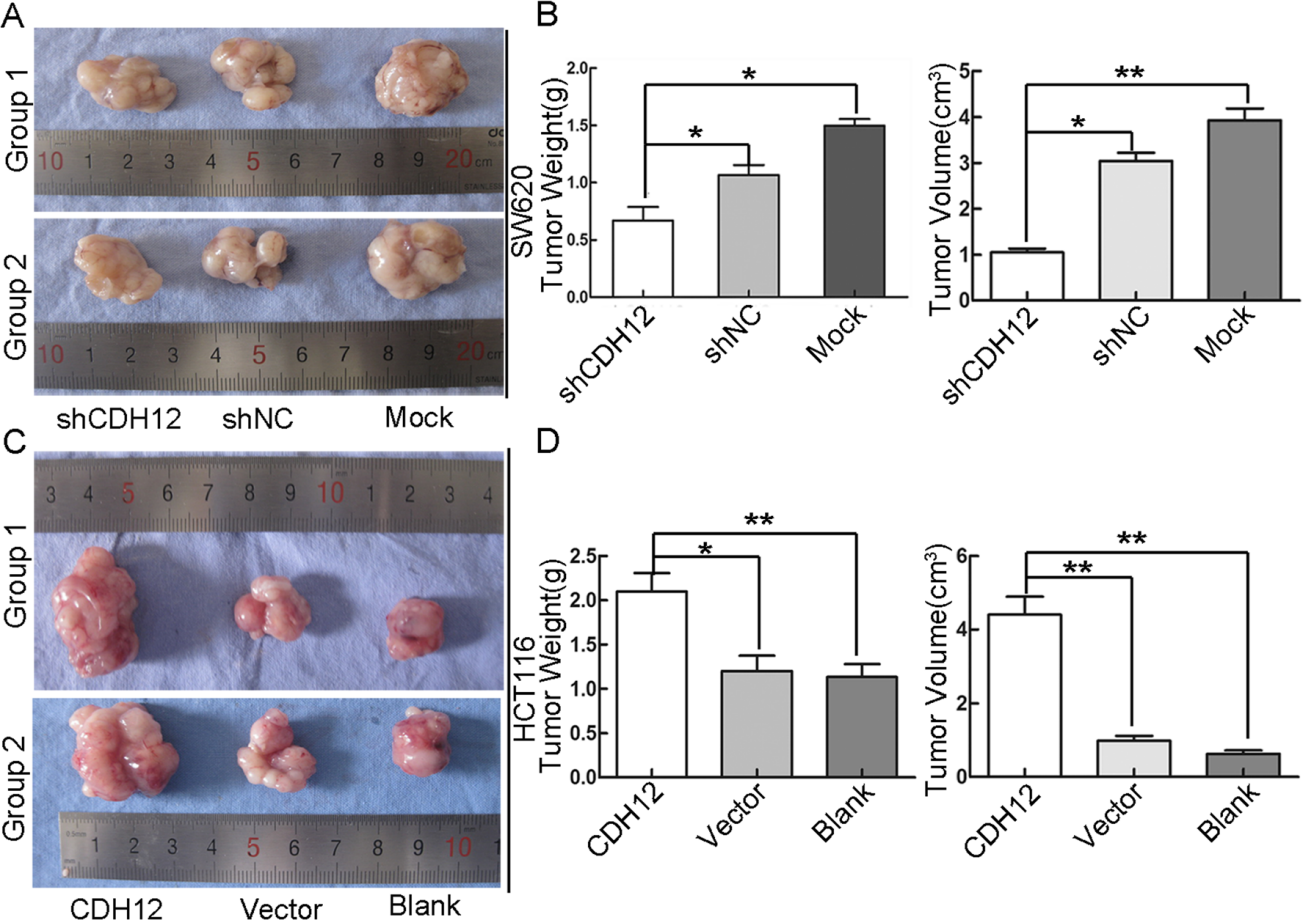
Effect of enforced/repressed CDH12 on tumorigenicity in nude mice. **a** Low expression of CDH12 in SW620 cells induces small nodules in nude mice. **b** *Column graph* indicates that the weight (*left panel*) and size (*right panel*) of tumor nodules in SW620/shCDH12 group are lower than control groups (*P < 0.05, **P < 0.01). **c** High expression of CDH12 promotes formation of tumor nodules in nude mice. **d** *Column graph* indicates that the weight of tumor nodules in HCT116/CDH12 group is heavier than control groups (*left panel*). The size of tumor nodules in HCT116/CDH12 group is larger than control group (*right panel*) (*P < 0.05, **P < 0.01)

To further elucidate the function of CDH12 on proliferation, we performed clone formation assay. Results showed that clone colony number formed in SW620/shCDH12 group is lower than control groups. At the same time, after enforcing CDH12 expression, HCT116/CDH12 cells acquired stronger ability to form clone colony (Fig. 3b). Conclusively, these data further indicated that CDH12 might be a positive regulator of proliferation in CRC cells (Fig. 4).

### Enforcing/repressing CDH12 on CRC cells promotes/suppresses tumorigenicity in nude mice

To elucidate function of CDH12 in tumor development in vivo, we have chosen nude mice to perform animal experiments. The experimental results were summarized in Supplementary Fig. 3. Compared with control groups, the size and weight of tumor nodules were markedly suppressed in CDH12 downregulation group (SW620/shCDH12) (Supplementary Fig. 3a, b). Similarly, in CDH12 overexpression group (HCT116/CDH12), size and weight of tumor formed in nude mice were significantly higher than that in control groups (Supplementary Fig. 3c, d).

### CDH12 promotes cell migration and invasion progression of CRC cells

**Fig. 5:**
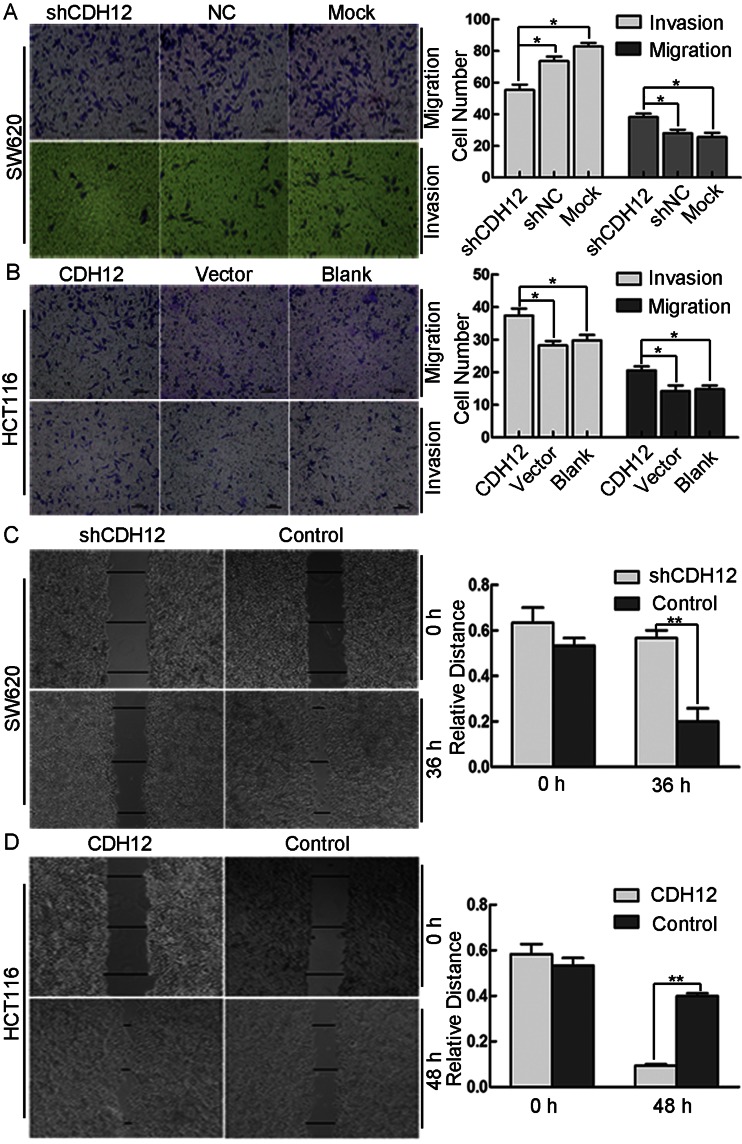
Transwell and wound-healing assay. **a**,**b** *left panel*, photograph of SW620 cell group (**a**) and HCT116 cell group (**b**) which migrate or invasion into the other side of the chamber in transwell assay; *right panel*, *column graph* shows that the number of migrated or invasive cells in SW620/shCDH12 or HCT116/CDH12 is less or more than their control group, respectively(*P < 0.05). **c**, **d** Wound-healing assay shows a significant decrease or increase in healing rate of the scramble wound in SW620/shCDH12 or HCT116/CDH12, respectively(**P < 0.01)

CDH12 belongs to cadherin family which acts as an important regulator of cancer cell migration. To detect the influence of CDH12 on migration and invasion in CRC cells, we performed transwell assay and found that SW620 cells with downregulated CDH12 expression presented decreased invasion and migration ability compared with the control groups (*P* < 0.05). Consistently, ectopic expression of CDH12 in HCT116 cells promoted the migration and invasion ability of CRC cells (*P* < 0.05) (Fig. 5a, b, left panel). Cell numbers were counted at random five views; the average number of migratory cells was shown in Fig. 5a, b (right panel). Moreover, wound-healing assay was also used to examine migration ability of SW620/shCDH12 and HCT116/CDH12. As shown in Fig. 5c, d, after 48-h incubation with serum-free medium, the distance of the scratch wound in SW620/shCDH12 group is significantly larger compared with control groups. But in HCT116/CDH12 group, the distance of the scratch wound increased significantly compared with control group (Fig. 5c, d, left panel). Three random lines were drawn in each group, and relative length was calculated which is shown in Fig. 5c, d (right panel).

### CDH12 promotes migration and invasion of CRC cells through induction of EMT

**Fig. 6:**
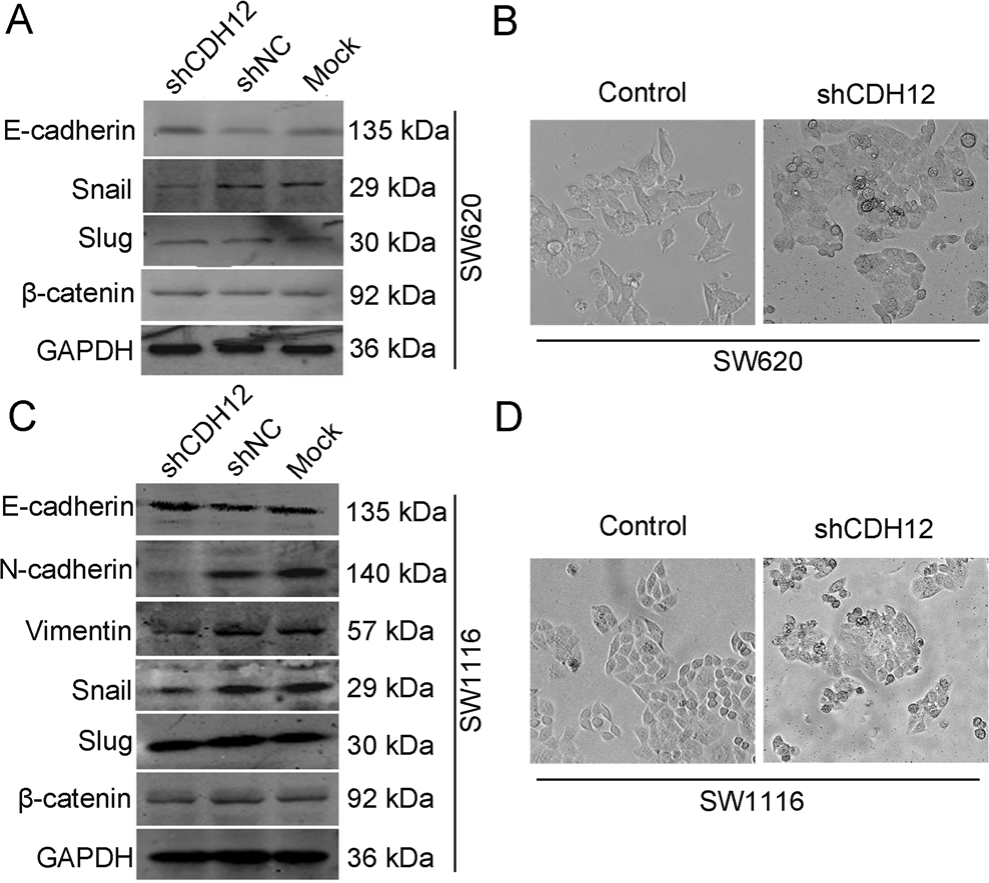
Downregulation of CDH12 inhibits EMT in SW620/shCDH12 and SW1116/shRNA CDH12 cells. **a**, **c** Change of EMT markers and transcriptional factors in SW620/shCDH12 cells and SW1116/shCDH12 cells. **b**, **d** The cellular morphology of SW620 and SW1116 cells with low CDH12 expression

**Fig. 7:**
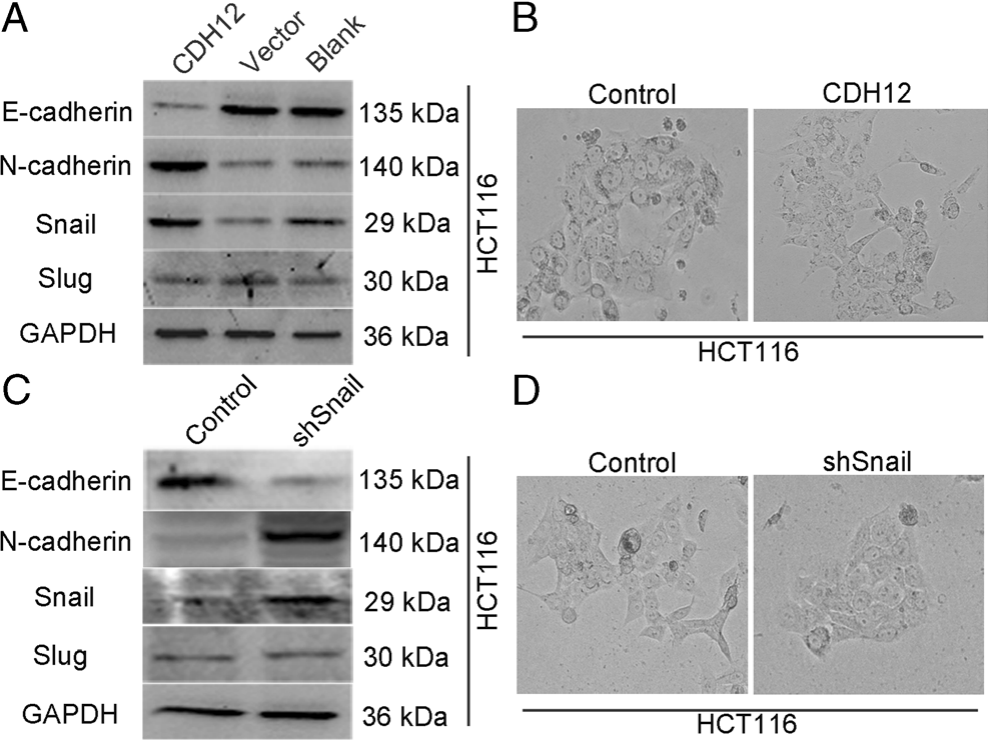
CDH12 induces EMT in HCT116 cells. **a** Change of EMT markers and transcriptional factors in HCT/CDH12 cells. **b** The cellular morphology of HCT116 cells with high CDH12 expression. **c** Change of EMT markers after interfering Snail expression in HCT116/CDH12 cells. **d** The cellular morphology of HCT116/CDH12 cells with low Snail expression

**Fig. 8:**
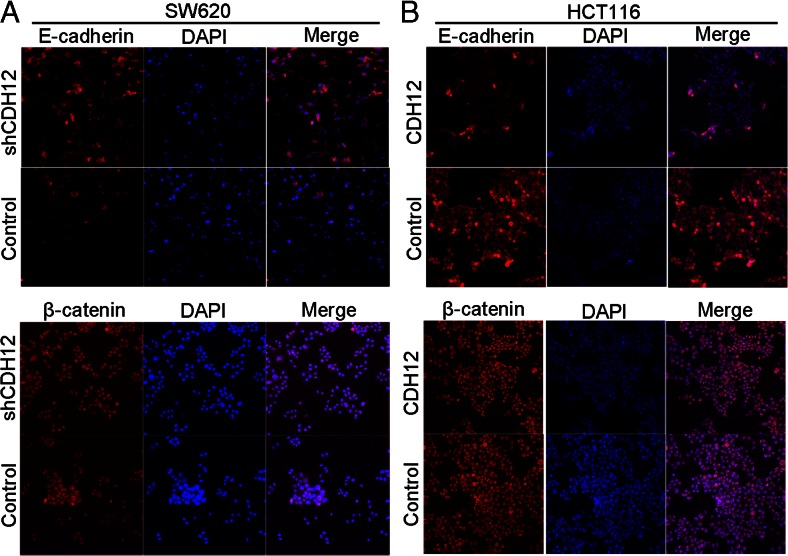
Immunofluorescent staining showed changes in EMT marker expression in SW620/shCDH12 and HCT116/CDH12. **a** E-cadherin expression is increased, and no change of β-catenin is observed in SW620/shCDH12 cells. **b** E-cadherin expression is decreased, and no change of β-catenin is observed in HCT116/CDH12 cells

The EMT of tumor cells is widely accepted to closely correlate with cancer metastasis. During this progress, endothelial cells expressing E-cadherin switch into mesenchymal cells marking with N-cadherin expression naming “cadherin switch” [15]. To explore whether CDH12 can promote CRC cell migration and invasion through induction of EMT, we firstly observed and compared morphology of SW620/shCDH12 and HCT116/CDH12 cells with their control group, respectively. As shown in Fig. 6b, SW620 cells expressing low CDH12 levels had a typical epithelium-like appearance not the mesenchymal phenotype which favors the metastasis of cancer cells. Whereas, HCT116/CDH12 cells with high CDH12 expression presented a fibroblastic phenotype (Fig. 7b). We then detected the change of EMT markers in SW620/shCDH12 and HCT116/CDH12; the downregulation of CDH12 led to high expression of E-cadherin in SW620/shCDH12 cells (Fig. 6a). Because we could not detect the expressions of vimentin and N-cadherin in SW620, we construct SW1116/shCDH12 cells to verify the change of EMT markers. It also showed upregulation of E-cadherin as well as downregulation of vimentin and N-cadherin in SW1116/shCDH12 cells (Fig. 6c) and the change of SW1116 cell phenotype (Fig. 6d). In addition, we found decreased expression of E-cadherin and increased expression of N-cadherin in HCT116/CDH12 cells as well as cell phenotype change (Fig. 7a, b). Immunofluence staining has also verified the downregulation of E-cadherin in SW620/shCDH12 cells and upregulation of E-cadherin in HCT116/CDH12 cells (Fig. 8).

To further characterize transcriptional factors involved in CRC EMT triggered by CDH12, we also detected the expressions of Snail and Slug in stably transfected cells versus parental cells by Western blot analysis. As shown in Fig. 6a, c, we found a significant downregulation of Snail in SW620/shCDH12 and SW1116/shCDH12 cells; in addition, high levels of Snail were found in HCT116/CDH12 cells (Fig. 7a). We did not found significant change in the expression of slug in these cells (Figs. 6a, c and 7a). This may partially indicate that Snail is required for EMT induced by CDH12. We next transfected shRNA-Snail cells into HCT116/CDH12 cells which expressed high level of Snail and examined the change of EMT markers. Expression of E-cadherin was enhanced, whereas expression of N-cadherin was downregulated in HCT116/CDH12/shRNA-Snail cells (Fig. 7c). These findings indicate that CDH12 may contribute to CRC cell metastasis by promoting EMT, and this progress is induced by targeting Snail.

### Expression of CDH12 in CRC cells is modulated by MCP1

MCP1 acts as a key CC chemokine and is responsible for trafficking and activation of monocytes/macrophages through its receptor CCR2 [16]. Recently, elevated MCP1 expression level in the tumor implicates that it is crucial for cancer growth, dissemination, and metastasis [17, 18]. MCPIP, which was originally found in human monocytes after treatment with MCP1, has been verified that it is able to combine with CDH12 gene and promote the expression of CDH12 [19]. Therefore, we used recombinant MCP1 to stimulate CRC cells and found that MCP1 was able to induce the expression of MCPIP and consistent high expression of CDH12 in a time-dependent manner in HCT116. The premium working point was 12 h (Supplementary Fig. 5b, d). In SW620 cell line, we used recombinant MCP1 and MCPIP small interfering RNA (siRNA) to detect the expression of CDH12. Results showed that recombinant MCP1 could induce consistent high expression of MCPIP and CDH12. In addition, MCPIP siRNA was able to block the MCPIP-induced expression of CDH12 which demonstrated that CDH12 expression was MCPIP dependent (Supplementary Fig. 5a, c). That means that MCP1 can promote CDH12 expression through induction of MCPIP.

## Discussion

Carcinogenesis of colorectal cancer is a multistep process including complicated molecular biological events influencing cell proliferation stability and metastasis potential [20]. Distant metastasis, especially liver metastasis, rather than the primary tumor from which these lesions originate, is responsible to the poor prognosis and high mortality of CRC patients [21–23]. More than 50–60 % CRC patients suffer from metastasis; liver is the most susceptible organ in this metastasis process, and more than 80–90 % liver metastasis are unresectable lesions [24, 25]. In our present study, we demonstrated that CDH12 acts as an oncogene in colorectal cancer cell invasion and migration. Moreover, this positive process is achieved by promoting EMT-targeting Snail.

CDH12 is a subtype of N-cadherin family expressing 88-kD protein. CDH12 can promote the migration and invasion abilities of salivary adenoid cystic carcinoma [11]. Farzana et al. reported an unusual expression of CDH12 in CRC tissue [26]. In our study, we performed immunohistochemistry staining in tissue microarray of 78 cases and further found that CDH12 expression was significantly increased in CRC tissues compared to adjacent normal tissue. In statistical analysis of clinical materials, we found that CDH12 high expression was associated with invasion depth of tumor tissue and lymph node lesions, independent of age, gender, tumor site, tumor histology, and tumor size. In addition, high expression of CDH12 predicted poor prognosis of CRC patients because these patients has a poorer OS and DFS compared with low CDH12 expression patients. These findings indicate the oncogenic role of CDH12 in CRC which is consistent with the findings previously.

To clearly reveal the role of CDH12 in CRC cell proliferation, we performed CCK-8 proliferation assay and clone formation assay in SW620 and HCT116 cells. We observed that downregulation of CDH12 significantly suppresses SW620 cell proliferation ability and ectopic expression of CDH12 promotes cell proliferation in HCT116. In addition, clone colony has a significant decrease/increase in SW620/shCDH12 and HCT116/CDH12 cell lines. Comparing with control group, our results showed that enforcing/repressing CDH12 on CRC cells can promote/suppress tumorigenicity in nude mice. Accordingly, our findings were supportive to promoting proliferation effect of CDH12.

To further verify the function of CDH12 in CRC cells, we performed transwell assay. Results showed suppressive role of downregulated CDH12 in CRC cell migration and invasion. Consistently, we also observed that enforcing CDH12 could promote CRC cell migration and invasion. These data indicate partially the important role of CDH12 in CRC cell metastasis which was in agreement with the findings in salivary adenoid cystic carcinoma [6]. Considering the important role of EMT in cancer cell invasion into vessels, during EMT, a tumor cell with epithelial characteristic transitions to tumor cell with mesenchymal characteristics through modulation of cell polarity and adhesion [3, 27], we investigated the influence of CDH12 in EMT markers. In our study, we found that CDH12 downregulation promoted the expression of epithelial marker E-cadherin and decreased the expression of mesenchymal markers Vimentin and N-cadherin; the similar results were also observed in CDH12 high-expression cell line, indicating that CDH12 could promote EMT. Moreover, we found significant change in the expression of Snail which functions in the process of downregulation of E-cadherin. The DNA-binding site of Snail is able to match the two E-boxes in E-cadherin promoters which exert the effect on downregulation of E-cadherin [28], and such a downregulation may have a causal role in cancer migration [29]. Accumulating evidences demonstrate the reverse correlation between Snail and E-cadherin in both human hepatocellular carcinoma (HCC) cells and tissues [30, 31], melanoma cells [32], and squamous cell carcinomas [33]. In our study, we also found this reverse correlation between Snail and E-cadherin. This indicates partially, but not completely, that CDH12 might promote EMT by activating transcriptional factor Snail.

MCP1 is a 76 amino acid peptide which acts as a key CC chemokine in tumor microenvironment. It can recruit monocytes and macrophages to inflammatory sites and regulate their activities [34]. Yoshimua et al. reported that stromal cell-derived MCP1 in primary tumors promoted metastasis of breast cancer cells to the lungs [35]. MCPIP, which was originally found in human monocytes after treatment with MCP1, has been verified that it is able to combine with CDH12 gene and enhanced endothelial cell apoptosis, proliferation, and migration [19]. Therefore, we detected the expressions of MCPIP and CDH12 after stimulating of MCP1 and found that MCP1 can induce MCPIP and CDH12 expressions. The elevated level of CDH12 in CRC cell is consistent with MCPIP, and the downregulation of MCPIP can also decrease the level of CDH12 protein. That means that MCP1 may act as an extracellular modulated molecule and is able to induce the expression of MCPIP by binding to relative receptor. MCPIP combines to the target CDH12 gene and promote the transcription of CDH12. Taken together, our study suggest that CDH12 might contribute to CRC cell metastasis via promoting EMT, and this process was accomplished by activating transcriptional factor Snail. Moreover, MCP1 may act as an upstream modulated molecule of CDH12.

In conclusion, we present convincing evidence showing that CDH12 is a prognostic factor in CRC patients and plays an oncogenic role in CRC cells and tumorgenicity in nude mice. Through activating transcriptional factor Snail, CDH12 regulates E-cadherin expression and promote the emergence of EMT which contributes to the metastasis of CRC cells. However, the role of CDH12 in CRC cell proliferation has not been fully understood and our findings only partially unveil the molecular mechanisms of CDH12 in EMT. Therefore, future studies are required to elucidate the function of CDH12 in CRC.

## Electronic supplementary material

Below is the link to the electronic supplementary material.

Supplementary Fig. 1 Immunohistochemical staining results of CDH12 in tumor tissues and adjacent normal tissue; Kaplan-Meier survival curves for OS and DFS. **a** Uniformly negative staining in adjacent normal mucosa, **b** heterogeneous staining in adjacent normal mucosa, **c** uniformly positive staining in adjacent normal mucosa, **d** uniformly negative staining in CRC tumor tissues, **e** heterogeneous staining in CRC tumor tissues, and **f** uniformly positive staining in CRC tumor tissues. Magnification 200×. **g** CDH12 high expression indicates undesirable OS and **h** CDH12 high expression indicates unfavorable DFS. *P* < 0.05, both in OS and DFS. (GIF 247 kb)

High-resolution image (TIF 12,330 kb)

Supplementary Fig. 2 Effect of enforced/repressed CDH12 on tumorigenicity in nude mice. **a** Low expression of CDH12 in SW620 cells induces small nodules in nude mice. **b**, **c** Column graph indicates that the weight (**b**) and size (**c**) of tumor nodules in SW620/shCDH12 group are lower than control groups (*P < 0.05, **P < 0.01). (GIF 113 kb)

High-resolution image (TIF 7853 kb)

Supplementary Fig. 3 Effect of enforced/repressed CDH12 on tumorigenicity in nude mice. **a** Low expression of CDH12 in SW620 cells induces small nodules in nude mice. **b** Column graph indicates that the weight (left panel) and size (right panel) of tumor nodules in SW620/shCDH12 group are lower than control groups (*P < 0.05, **P < 0.01). **c** High expression of CDH12 promotes formation of tumor nodules in nude mice. **d** Column graph indicates that the weight of tumor nodules in HCT116/CDH12 group is heavier than control groups (left panel). The size of tumor nodules in HCT116/CDH12 group is larger than control group (right panel) (*P < 0.05, **P < 0.01). (GIF 109 kb)

High-resolution image (TIF 8783 kb)

Supplementary Fig. 4 Liver metastatic lesions of CRC cell line HCT116. HCT116 with high-expression CDH12 tends to form distant liver colonization (**a**, black arrow) compared with control group (**b**) in intrasplenic injection nude model. (GIF 150 kb)

High-resolution image (TIF 8405 kb)

Supplementary Fig. 5 Western blot analysis. **a**, **c** Recombinant MCP1 can induce consistent high expressions of MCPIP and CDH12. In addition, MCPIP siRNA was able to block the MCPIP-induced expression of CDH12 which demonstrated that CDH12 expression was MCPIP dependent. **b**, **d** MCP1 is able to induce the expression of MCPIP and consistent high expression of CDH12 in a time-dependent manner in HCT116. The premium working point was 12 h. (GIF 53 kb)

High-resolution image (TIF 7654 kb)

## Abbreviations

CDH12: Cadherin-12
CRC: Colorectal carcinoma
EMT: Epithelial-mesenchymal transition
BCA: Bicinchonininc acid
