# Supplementary figures and images for: Cadherin-12 enhances proliferation in colorectal cancer cells and increases progression by promoting EMT
Source: Tumour Biol. 2016 Jan 14;37(7):9077–88. doi: 10.1007/s13277-015-4555-z (PMC4990612; doi:10.1007/s13277-015-4555-z)

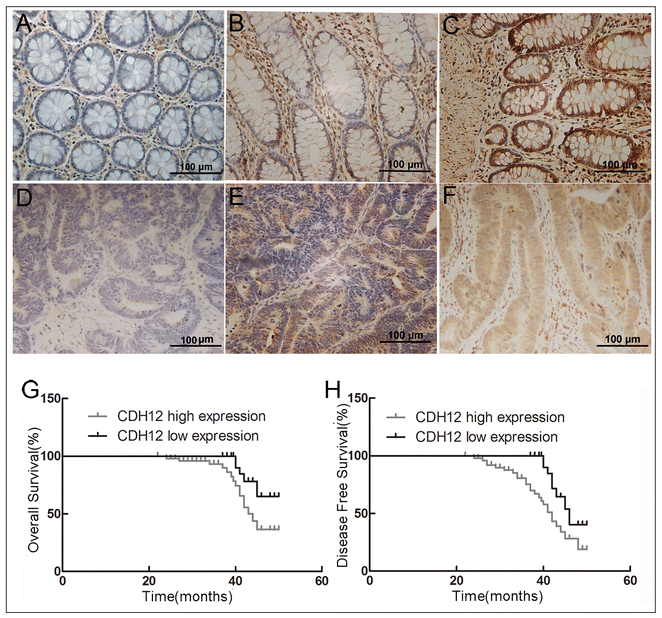

Supplement: Supplementary file 1 — Immunohistochemical staining results of CDH12 in tumor tissues and adjacent normal tissue; Kaplan-Meier survival curves for OS and DFS. a Uniformly negative staining in adjacent normal mucosa, b heterogeneous staining in adjacent normal mucosa, c uniformly positive staining in adjacent normal mucosa, d uniformly negative staining in CRC tumor tissues, e heterogeneous staining in CRC tumor tissues, and f uniformly positive staining in CRC tumor tissues. Magnification 200×. g CDH12 high expression indicates undesirable OS and h CDH12 high expression indicates unfavorable DFS. P < 0.05, both in OS and DFS. (GIF 247 kb) [file 13277_2015_4555_Fig9_ESM.gif]

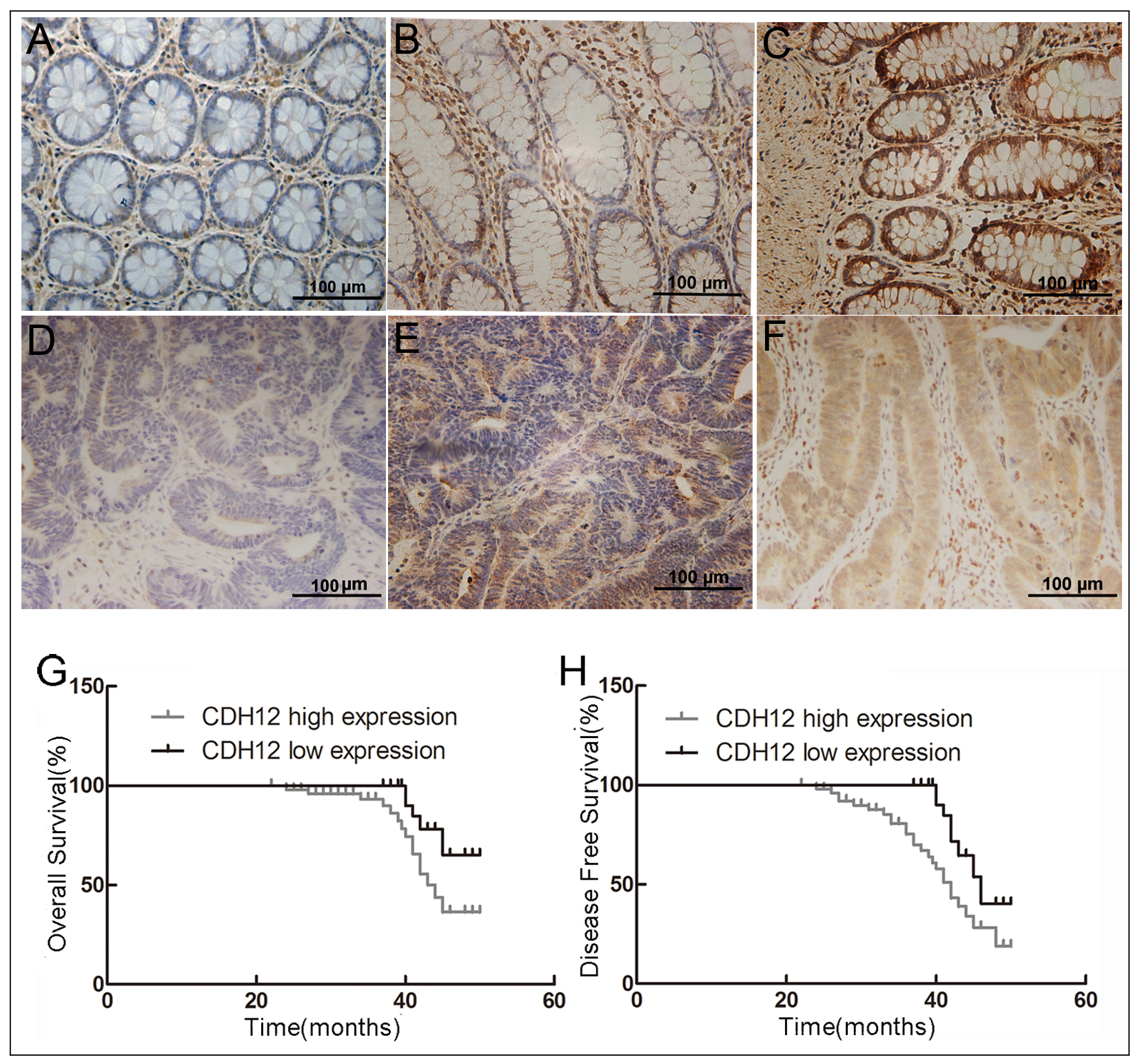

Supplement: Supplementary file 2 — High-resolution image (TIF 12,330 kb) [file 13277_2015_4555_MOESM1_ESM.tif]

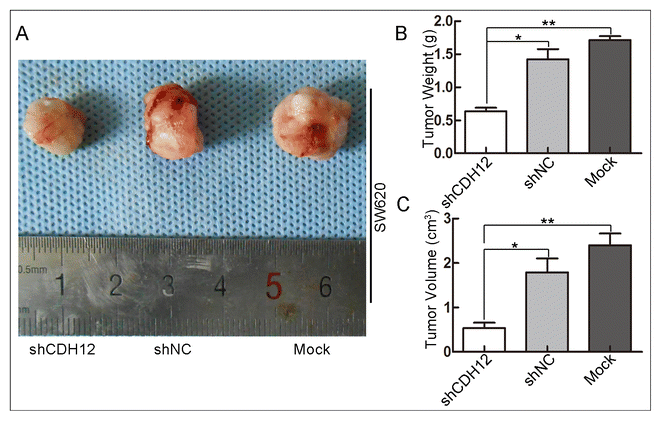

Supplement: Supplementary file 3 — Effect of enforced/repressed CDH12 on tumorigenicity in nude mice. a Low expression of CDH12 in SW620 cells induces small nodules in nude mice. b, c Column graph indicates that the weight (b) and size (c) of tumor nodules in SW620/shCDH12 group are lower than control groups (*P < 0.05, **P < 0.01). (GIF 113 kb) [file 13277_2015_4555_Fig10_ESM.gif]

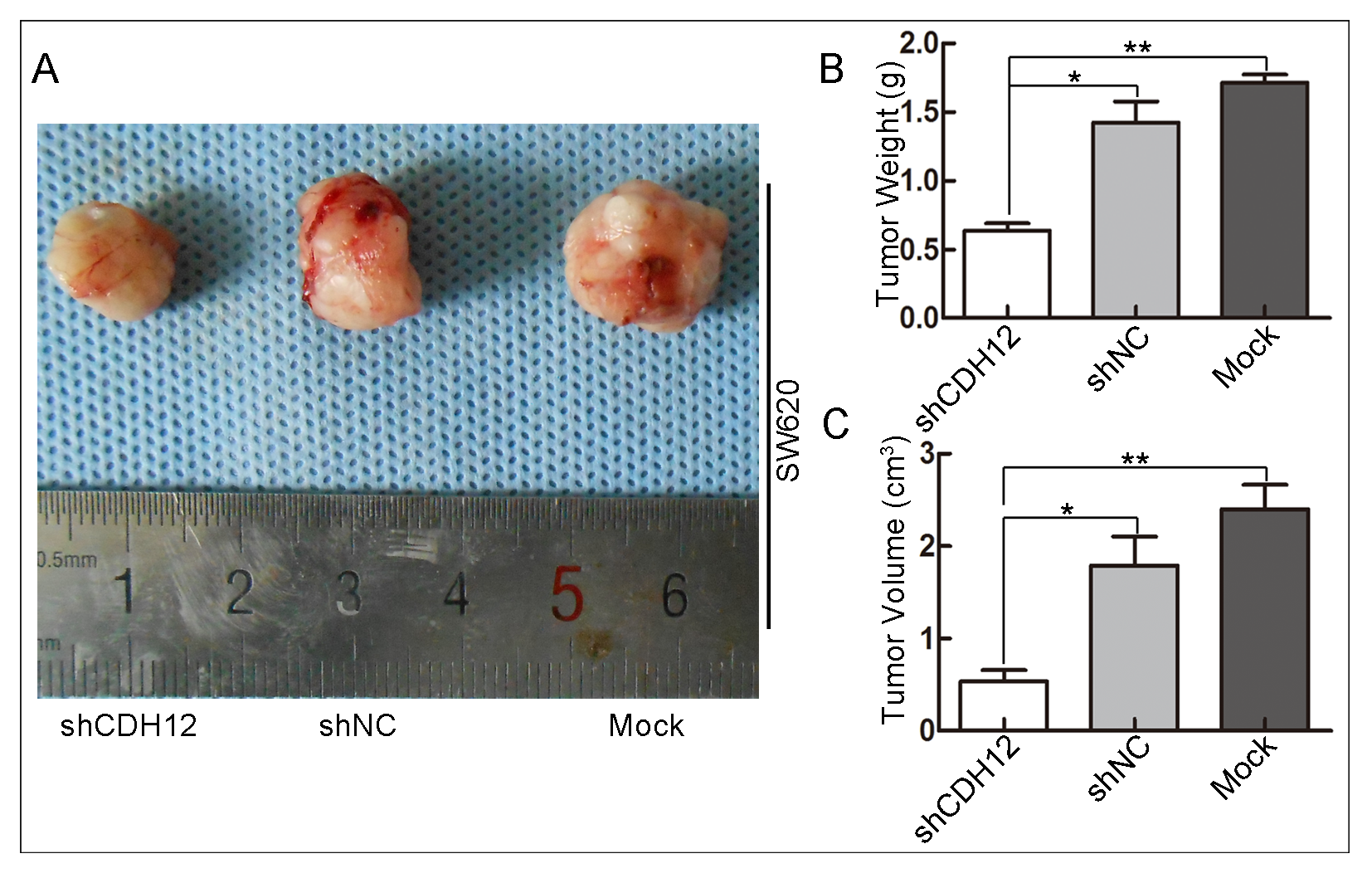

Supplement: Supplementary file 4 — High-resolution image (TIF 7853 kb) [file 13277_2015_4555_MOESM2_ESM.tif]

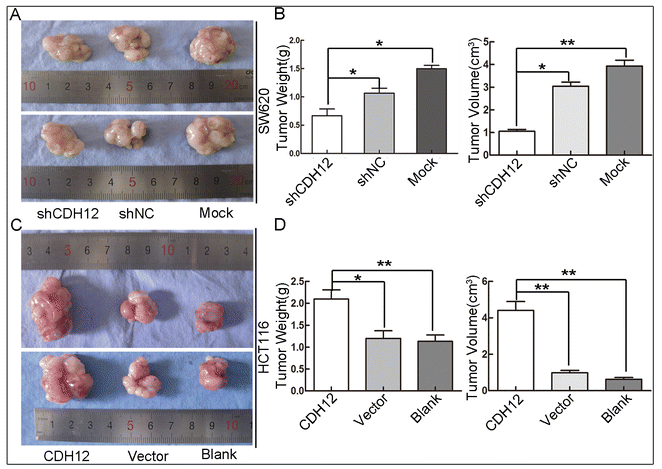

Supplement: Supplementary file 5 — Effect of enforced/repressed CDH12 on tumorigenicity in nude mice. a Low expression of CDH12 in SW620 cells induces small nodules in nude mice. b Column graph indicates that the weight (left panel) and size (right panel) of tumor nodules in SW620/shCDH12 group are lower than control groups (*P < 0.05, **P < 0.01). c High expression of CDH12 promotes formation of tumor nodules in nude mice. d Column graph indicates that the weight of tumor nodules in HCT116/CDH12 group is heavier than control groups (left panel). The size of tumor nodules in HCT116/CDH12 group is larger than control group (right panel) (*P < 0.05, **P < 0.01). (GIF 109 kb) [file 13277_2015_4555_Fig11_ESM.gif]

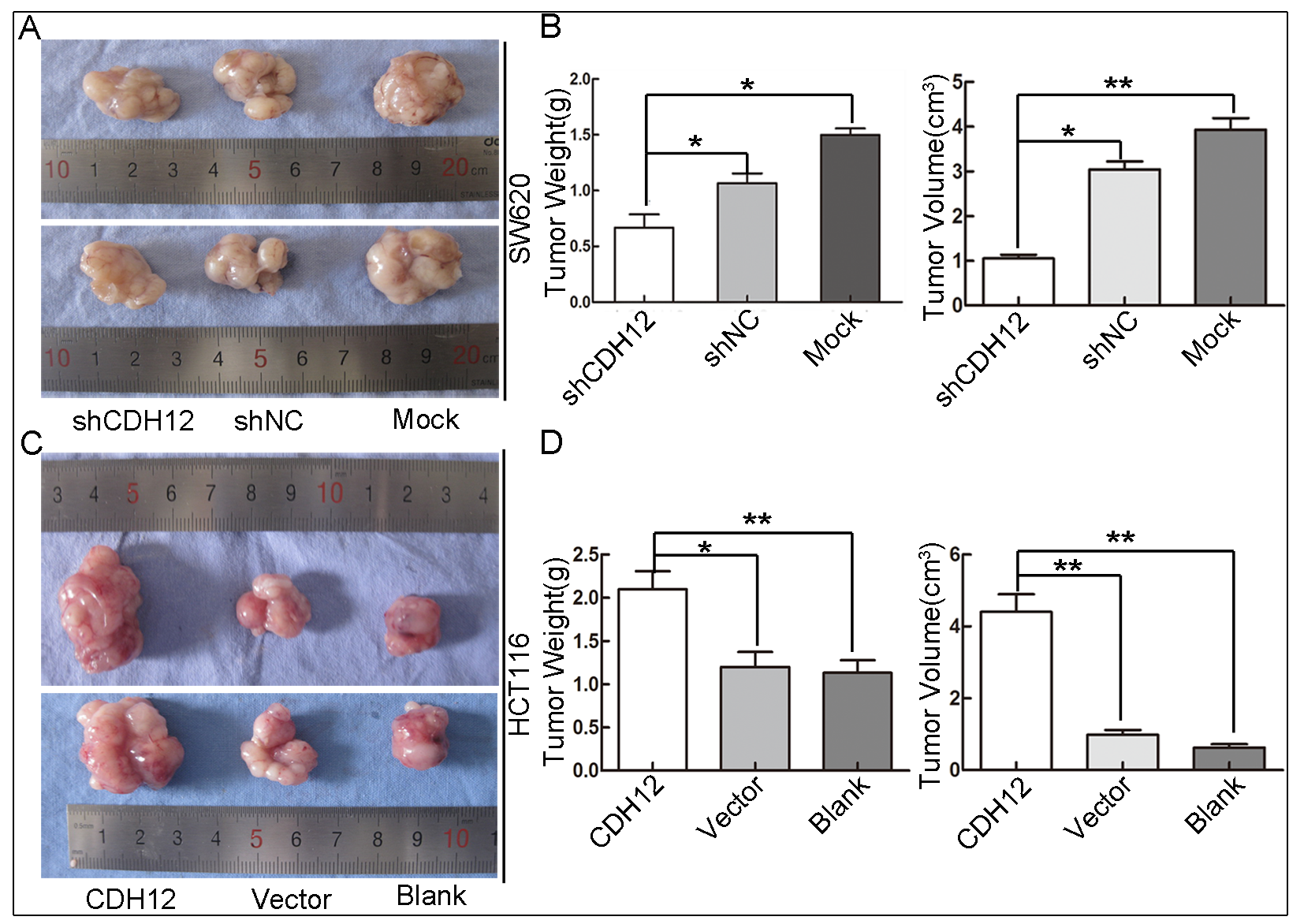

Supplement: Supplementary file 6 — High-resolution image (TIF 8783 kb) [file 13277_2015_4555_MOESM3_ESM.tif]

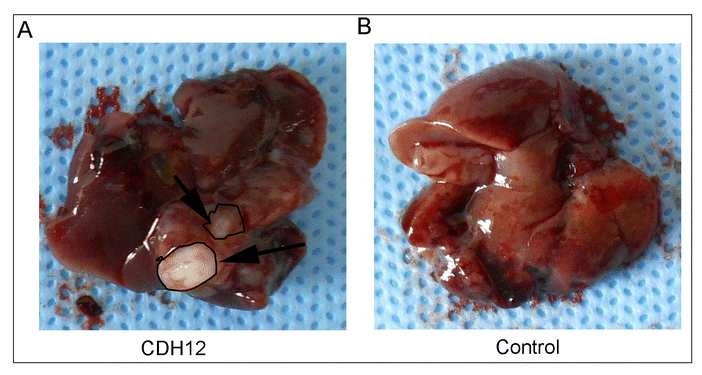

Supplement: Supplementary file 7 — Liver metastatic lesions of CRC cell line HCT116. HCT116 with high-expression CDH12 tends to form distant liver colonization (a, black arrow) compared with control group (b) in intrasplenic injection nude model. (GIF 150 kb) [file 13277_2015_4555_Fig12_ESM.gif]

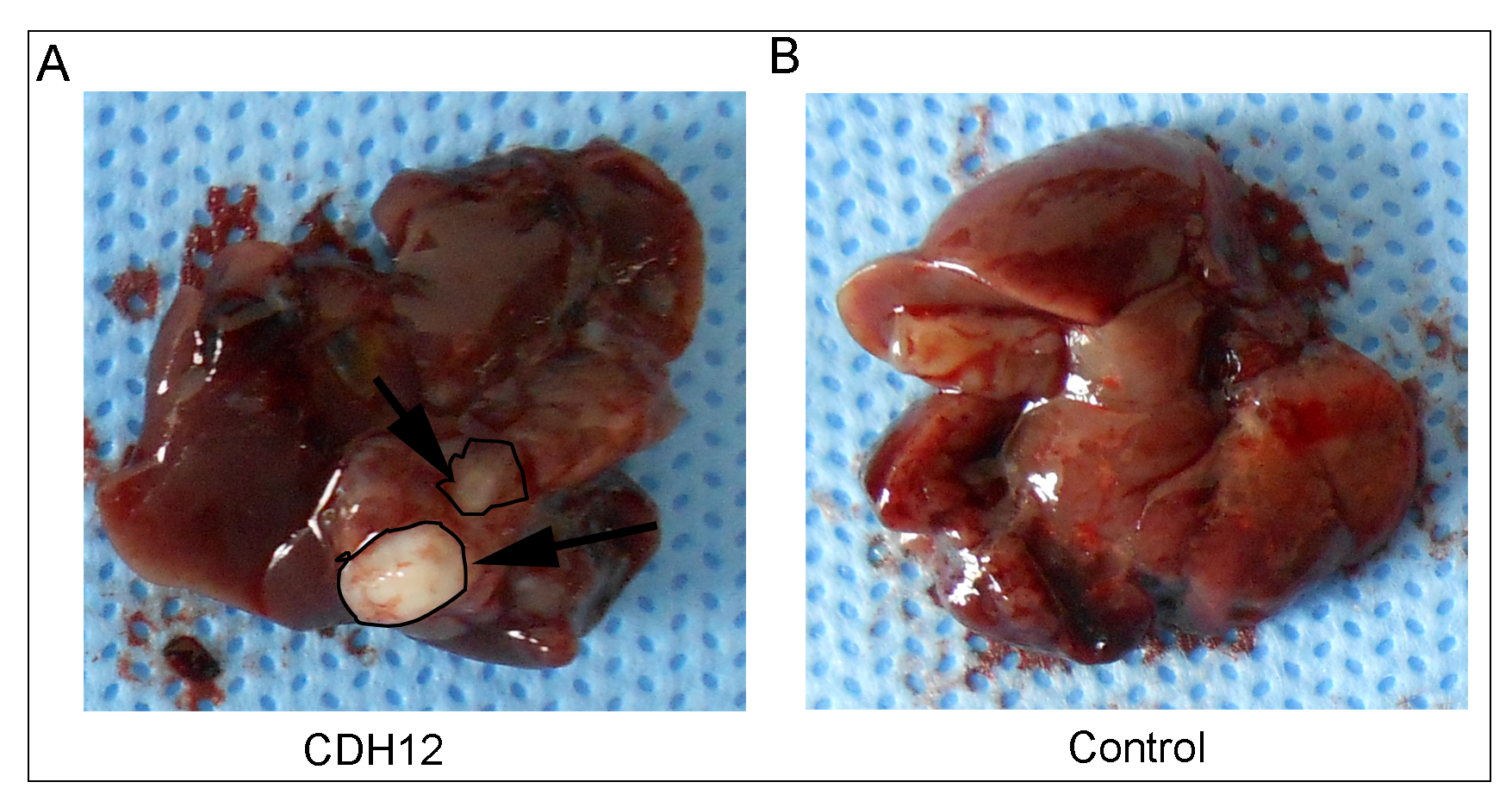

Supplement: Supplementary file 8 — High-resolution image (TIF 8405 kb) [file 13277_2015_4555_MOESM4_ESM.tif]

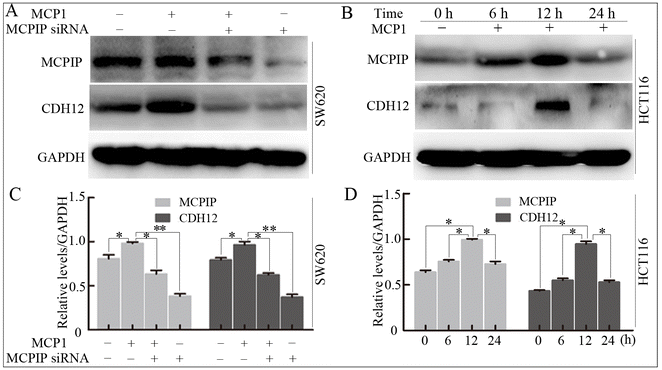

Supplement: Supplementary file 9 — Western blot analysis. a, c Recombinant MCP1 can induce consistent high expressions of MCPIP and CDH12. In addition, MCPIP siRNA was able to block the MCPIP-induced expression of CDH12 which demonstrated that CDH12 expression was MCPIP dependent. b, d MCP1 is able to induce the expression of MCPIP and consistent high expression of CDH12 in a time-dependent manner in HCT116. The premium working point was 12 h. (GIF 53 kb) [file 13277_2015_4555_Fig13_ESM.gif]

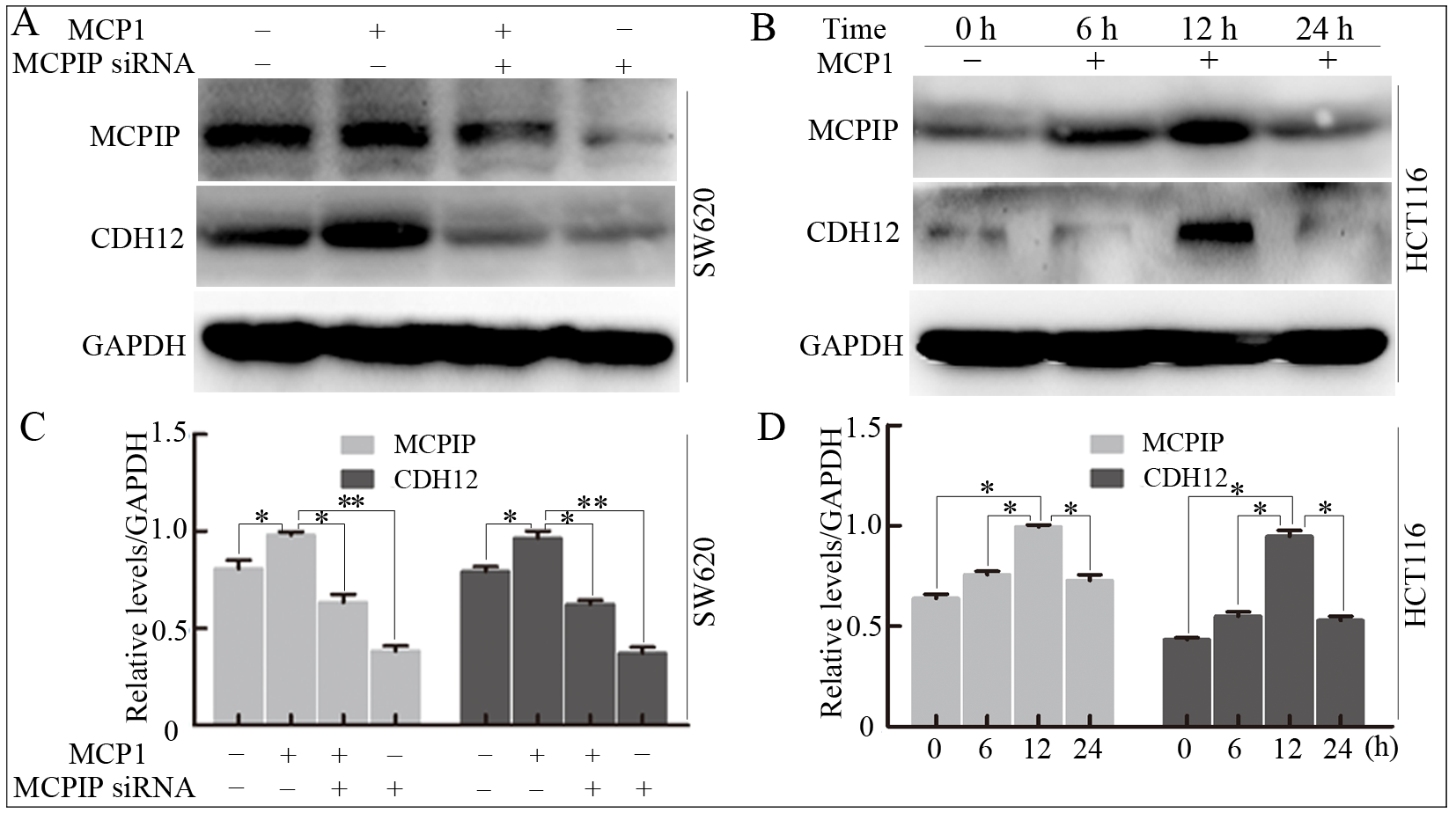

Supplement: Supplementary file 10 — High-resolution image (TIF 7654 kb) [file 13277_2015_4555_MOESM5_ESM.tif]
